# Cost-effectiveness and budget impact analysis of enzalutamide in comparison to abiraterone in treatment of metastatic prostate cancer resistant to castration in Iran

**DOI:** 10.1186/s12894-024-01431-w

**Published:** 2024-02-20

**Authors:** Zahra Goudarzi, Farhad Lotfi, Zhila Najafpour, AliAkbar Hafezi, Marzieh Alizadeh Zakaria, Khosro Keshavarz

**Affiliations:** 1 Health Human Resources Research Center, School of Health Management and Information Sciences, Shiraz University of Medical Sciences, Shiraz, Iran; 2 Emergency Medicine Research Center, Shiraz University of Medical Sciences, Shiraz, Iran; 3 Department of Health care Management, School of Public Health, Ahvaz Jundishapur University of Medical Sciences, Ahvaz, Iran; 4 Department of Radiation Oncology, School of Medicine, Shiraz University of Medical Sciences, Shiraz, Iran; 5 Student Research Committee, School of Management and Medical Information Sciences, Shiraz University of Medical Sciences, Shiraz, Iran; 6 Health Human Resources Research Center, Department of Health Economics, School of Health Management and Information Sciences, Shiraz University of Medical Sciences, Shiraz, Iran

**Keywords:** Cost-effectiveness, Enzalutamide, Prostate cancer resistant to castration, Abiraterone

## Abstract

**Introduction:**

In recent years, enzalutamide and abiraterone have been widely used as treatments for metastatic castration-resistant prostate cancer (mCRPC). However, the cost-effectiveness of these drugs in Iran is unknown. This study evaluated the cost-effectiveness of enzalutamide for the treatment of metastatic prostate cancer resistant to castration in Iran.

**Methods:**

A 3-state Markov model was developed to evaluate the cost-effectiveness of enzalutamide and abiraterone from a social perspective over 10 years. The clinical inputs were obtained from the meta-analysis studies. The direct medical costs were obtained from the tariffs of the healthcare system, while the direct non-medical and indirect costs were collected from the patients. The data of utilities were derived from the literature. In addition, sensitivity analyses were conducted to assess the uncertainties.

**Results:**

**C**ompared with Abiraterone, enzalutamide was associated with a high incremental cost-effectiveness ratio (ICER) of $6,260 per QALY gained. According to the one-way sensitivity analysis, ICER was most heavily influenced by the prices of enzalutamide and Abiraterone, non-medical costs, and indirect costs. Regardless of the variation, enzalutamide remained cost-effective. The budget impact analysis of enzalutamide in the health system during 5 years was estimated at $6,362,127.

**Conclusions:**

At current prices, adding enzalutamide to pharmaceutical lists represents the cost-effective use of the healthcare resources in Iran for the treatment of metastatic castration-resistant prostate cancer.

## Introduction

Prostate cancer is the second most common cancer in men worldwide [1]. According to the GLOBOCAN report, 14,090 new cases of cancer occur in the world each year and the annual rate of death from this disease in 8,202 people around the world. In Iran, the cancer registry data showed that prostate cancer incidence in men was 9.11 per 100,000 people in 2018 [2]. Advanced prostate cancer has a poor prognosis and is difficult to treat. It can turn into castration-resistant prostate cancer (CRPC) within 1–2 years and easily progress to metastatic CRPC (mCRPC) [3].

Although the patients with localized prostate cancer are managed using radical surgery or radiation therapy, those with advanced or metastatic prostate cancer can initially be treated with androgen deprivation therapy (ADT) [4]. However, according to the results of a systematic review, 10–20% of the prostate cancer patients progressed to CRPC within 5 years. Metastatic CRPC (mCRPC) accounts for approximately 84% of these cases [5]. In recent years, the introduction of various treatment methods such as Sipuleucel-T, Cabazitaxel, abiraterone, enzalutamide, and radium-223 has increased the survival of cancer patients [6–9]. Once the dependence of prostate cancer on the androgen receptor pathway was identified, an incentive was provided to develop targeted therapies for extra-gonadal androgen signaling [10].

For mCRPC patients, taxane-based chemotherapy has been the treatment of choice for over a decade, since the success of the TAX327 trial [11]. Nevertheless, almost all patients with mCRPC develop drug resistance and eventually die within two or three years after the treatment with systemic chemotherapy [12].

abiraterone is a CPY17 enzyme inhibitor that inhibits residual androgen synthesis after androgen deprivation therapy and can be used to treat mCRPC in patients who have previously received chemotherapy [13, 14]. Enzalutamide, an oral drug that targets the androgen receptor-signalling pathway, can competitively inhibit androgen receptor binding. Compared to anti-androgens, such as bicalutamide, previously used in clinical therapy, enzalutamide has a 5- to 8-fold affinity for the androgen receptor [15, 16].

Several key phase III randomized controlled trials (RCTs) have shown that these new drugs significantly improve the survival of mCRPC patients either before or after chemotherapy [17]. However, various AR-targeting drugs, including abiraterone acetate, Enzalutamide, and Ureteronel (TAK-700), have shown conflicting therapeutic effects on oncologic outcomes in patients with mCRPC [18]. On the other hand, although these drugs changed the pattern of treatment for mCRPC patients, they have increased health cost. In the United States predicted that the cost of treating the patients would increase from $9.9 billion in 2006 to $15.41 billion in 2020 [19, 20]. Therefore, these drugs, which are effective but costly in different societies, can bring abought the challenge of cost-effectiveness. Considering this challenge, several studies evaluated the economic dimension of different therapeutic combinations containing abiraterone enzalutamide for mCRPC patients [21, 22].

Like many other countries, Iran is experiencing an increase in healthcare financial costs due to the high cost of medical technology. Therefore, the present study was conducted with the aim of evaluating the cost-effectiveness of enzalutamide versus abiraterone for mCRPC patients in the Iranian health system.

## Method

### Model structure

**Fig. 1:**
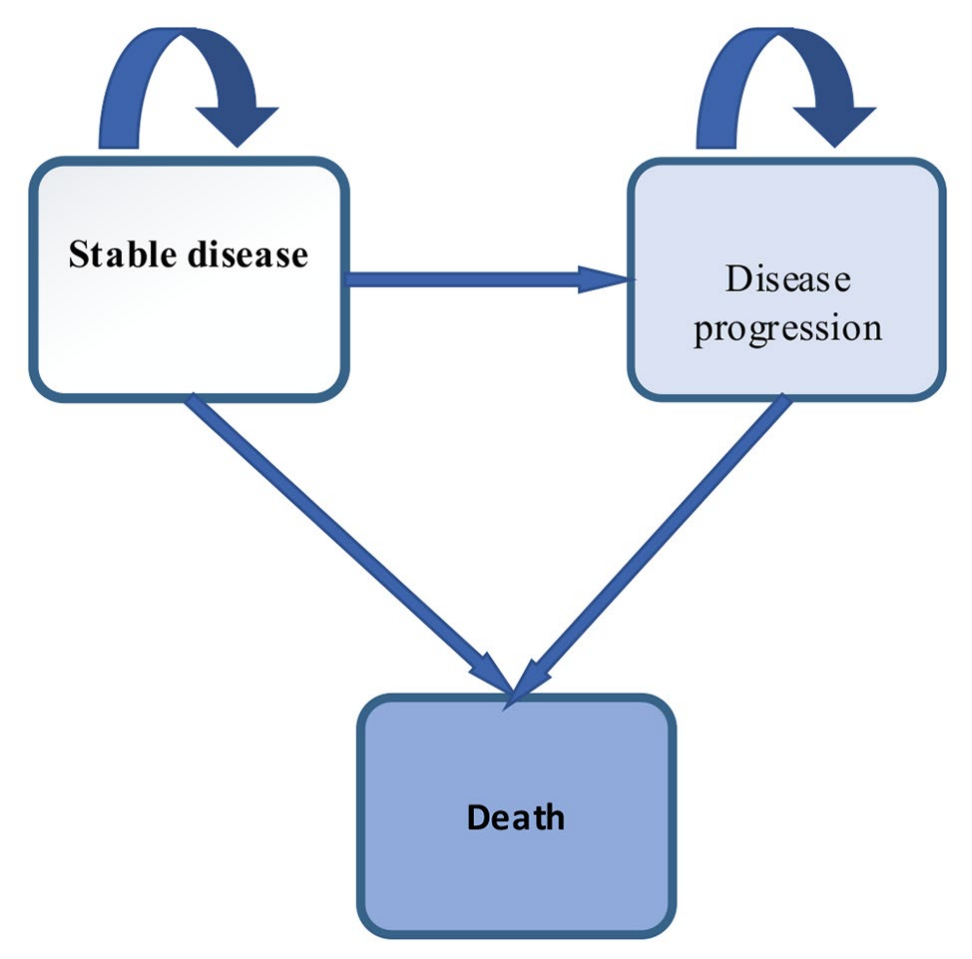
Markov model

This study used the Markov model, which includes three health states: non-progressive disease, progressive disease, and death (Fig. 1). For each of these health states, cost and utility were assigned in each model cycle to estimate cumulative costs and cumulative quality of life (QALYs) over the modeled time horizon. The analysis was done from the social perspective in the 2020 Treeage software. The length of the monthly cycle was used, and a time horizon of 10 years was chosen for the model. The costs and utilities were discounted at 7.2% and 3% per year, respectively. In order to evaluate the final result of the ICER study, it was compared with the threshold, which was one-time per capita GDP (equal to $18,261) [23].

### Patients and treatment

A hypothetical cohort population of 10,000 was modeled in this microsimulation study. The patients with histologically confirmed metastatic prostate cancer who had previously been treated with three or more courses of docetaxel and had disease progression were included.

These patients were treated with Enzalutamide, 160 mg daily, or abiraterone (1000 mg daily) with prednisolone (10 mg daily) until disease progression, death, or unacceptable toxicity. All of the patients with a serum testosterone level of 50 ng/dL (≤ 2.0 mmol/L) and adequate liver, kidney, and blood functions could be treated with the regimens listed, except for those who had bilateral orchiectomy. Moreover, the patients with hypersensitivity to enzalutamide and abiraterone or other components of the formulation, the presence of other malignancies (with the exception of basal cell carcinoma of the skin that was properly treated), or serious comorbidities not adequately controlled by other ongoing treatments (e.g., liver disease, diabetes, infection, heart disease, etc.) were not eligible for the study.

### Clinical efficacy

We conducted a systematic review of the clinical trial studies focusing on the analysis and comparison of the effectiveness of abiraterone and enzalutamide treatments. The records were found in PubMed, Scopus, and Web of Sciences databases using the following keywords: Enzalutamide, Abiraterone, Prostate Cancer, and randomized controlled trials. Unfortunately, no clinical trial study comparing enzalutamide and abiraterone directly was found. Therefore, the efficacy and survival probability data were extracted from the most recent meta-analysis study that directly focused on the comparison of enzalutamide and Abiraterone. The required efficacy data were progression-free survival and overall survival. In addition, the side effects of taking both drugs were included in the model. Given that the treatment rates were estimated based on annual amplitudes while the study cycles were monthly, all the data were converted to monthly probabilities.

### Cost and resource data

The cost of the model was estimated from a social perspective in Iran. The estimated costs per patients are presented in Table 1. The costs of diagnostic and laboratory tests, visits, chemotherapy, treatments related to adverse event (include anaemia, backpain, bone pain, diarrhoea, neutropenia), and other necessary supportive treatments were included in the model. The type of medical and therapeutic services and the frequency used in patients with mCRPC were obtained by reviewing reliable scientific sources and interviewing several experts in the field of urology and oncology [24, 25]. The drug prices were also obtained from https://irc.fda.gov.ir/nfi, an online resource that lists drug product pricing in Iran. Furthermore, the prices of other medical services were extracted from the tariffs approved by the Ministry of Health in April 2022. The direct non-medical costs were obtained by asking the patients. Direct non-medical costs included the costs of transportation, accommodation and food attributable to the disease. Indirect costs included the loss of productivity due to an absence from the workforce including early retirement and premature death. In addition, the human capital approach was used to calculate the indirect costs (lost income), assuming that the monetary value of lost production due to disability or death of a person was equal to the person’s salary before disability and death. Only the income loss and mortality costs of patients below the age of 65 were included in the calculation for indirect costs. The PPP was used to convert the rate of Rials to Dollars, where each dollar was equal to 30,697 Rials in 2022 [26].

**Table 1:** Input parameters for cost-effectiveness analysis

Variables	Value	Range	Distribution	Reference
**Clinical event probabilities (monthly)**				
**Progression disease**				
abiraterone group	0.0047	0.00423–00517	beta	[27]
enzalutamide group	0.0024	0.00216-.00264	beta	
**Death in progression state**				
abiraterone group	0.0019	0.00171-.00209	beta	[27]
enzalutamide group	0.0021	0.00189-.0023	beta	
**Death in progression free survival state**				
abiraterone group	0.009	0.0081-.0099	beta	[27]
enzalutamide group	0.008	0.0072-.0088	beta	
**AE** ^*^ **in abiraterone group (monthly)**				
Anemia	0.007	0.006-.008	beta	[28]
Backpain	0.033	0.028-.038	beta	[28]
Bone pain	0.084	0.071-.097	beta	[29]
Diarrhea	0.018	0.015-.021	beta	[28]
Fatigue	0.05	0.043-.058	beta	[28]
Neutropenia	0.003	0.0026-.0035	beta	[28]
Vomiting	0.022	0.019-.025	beta	[28]
**AE in enzalutamide group (monthly)**				
Anemia	0.005	0.004-.006	beta	[30, 31]
Backpain	0.084	0.071-.097	beta	[30, 31]
Arthralgia	0.017	0.014-.02	beta	[30, 31]
Diarrhea	0.013	0.011-.015	beta	[30, 31]
Fatigue	0.033	0.028-.038	beta	[30, 31]
vomiting	0.02	0.017-.023	beta	[30, 31]
**Utility**				
Progression-free	0.61	0.55-.68	beta	[32]
progression	0.31	0.28-.35	beta	[32]
Disutility anemia	0.11	0.099-.12	beta	[32]
Disutility backpain	0.06	0.054-.066	beta	[32]
Disutility bone pain	0.06	0.054-.066	beta	[32]
Disutility diarrhoea	0.2	0.18-.22	beta	[32]
Disutility fatigue	0.47	0.42-.52	beta	[32]
Disutility neutropenia	0.1	0.09-.12	beta	[32]
Disutility arthralgia	0.041	0.035-.047	beta	[33]
Disutility vomiting	0.09	0.07-.104	beta	[33]
**Cost ($)**				
enzalutamide	24$	19.2–28.8	gamma	https://irc.fda.gov.ir/nfi
abiraterone	13$	10.4–15.6	gamma	https://irc.fda.gov.ir/nfi
Laboratory tests	319$	255–386	gamma	https://treatment.tums.ac.ir
Imaging	213$	170–255	gamma	https://treatment.tums.ac.ir
Bone protecting agent	3,400$	2,720-4,080	gamma	https://treatment.tums.ac.ir
Radiopharmaceutical	6,853$	5,482-8,223	gamma	https://treatment.tums.ac.ir
Hospitalization	1,763$	1,410-2,115	gamma	https://treatment.tums.ac.ir
Chemotherapy	10,287 $	8,229 − 12,324	gamma	https://treatment.tums.ac.ir
Anemia	166$	141–191	gamma	https://treatment.tums.ac.ir
Backpain	84$	71–95	gamma	https://treatment.tums.ac.ir
Bone pain	95$	81–109	gamma	https://treatment.tums.ac.ir
Diarrhea	52$	44–60	gamma	https://treatment.tums.ac.ir
Fatigue	131$	111–151	gamma	https://treatment.tums.ac.ir
Neutropenia	154$	131–177	gamma	https://treatment.tums.ac.ir
Vomiting	85$	72–98	gamma	https://treatment.tums.ac.ir
**Direct non-medical cost**				
enzalutamide group	1,549 $	1,239-1,858	gamma	Interview
abiraterone group	2,132 $	1,705-2,558	gamma	
**Indirect cost**				
enzalutamide group	5,179 $	4,143-6,214	gamma	Interview
abiraterone group	6,906 $	5,524-8,287	gamma	

AE: Adverse Event

### Sensitivity analysis

The robustness of the results was analysed using the one-way sensitivity analysis in the form of a Tornado diagram and the one-way sensitivity analysis diagram, and the probabilistic sensitivity analysis in the form of ICER scatter plot diagrams. In the one-way sensitivity analysis, the cost parameters were variable at 20% efficiency and the utility and probability parameters were variable at 10% efficiency. In addition, in the probabilistic sensitivity analysis based on Monte Carlo simulation with 10,000 hypothetical patients, gamma distribution was used for cost inputs and beta distribution was used for probability values and utility.

### Budget impact analysis

A Markov-based model was applied to estimate the budget impact of enzalutamide for the treatment of high-risk mCRPC patients in the Iranian health program. The only comparator was Abiraterone. The budget impact analysis included the treatment costs for mCRPC and the treatment after progression to mCRPC.

The size of the treated population was estimated using the epidemiological data in Iran. According to the literature, the number of prostate cancer patients in the country was 16,071 in 2017 [34]. In addition, according to reliable scientific sources, the incidence rate of prostate cancer was 2 per 10,000 people in Iran [34]. Among all the patients with prostate cancer, there was a 20%-chance of developing castration-resistant metastatic [35] and mortality rate for mCRPC is 57% as found in the earlier studies [36].

## Results

The total costs over a 10-year time horizon were higher in the enzalutamide group than in the abiraterone ($17,541 vs. $16,408), predicting an incremental cost of $1,133. However, the total QALYs gained in the enzalutamide group were greater than in the other (1.02 versus 0.84), estimating an incremental QALY of 0.18.

The ICER obtained from the results of our study was 6,260 QALY/$, which was lower than the threshold of 18,261 dollars and indicated that enzalutamide was cost-effective in mCRPC patients compared to abiraterone (Table 2).

**Table 2:** Cost-effectiveness analysis results

strategy	cost	effect	Incr cost	Incr effect	ICER
enzalutamide	17,541	1.02	1133	0.18	**6,260**
abiraterone	16,408	0.84	-	-	-

### Sensitivity analysis

The results of the one-way sensitivity analysis conducted in the population of the MCRPC patients showed that the price of enzalutamide was the most influential parameter, but it did not have a significant effect on the results of the study, because it was still below the threshold. The price of Abiraterone, the direct non-medical costs, and the indirect costs had the greatest effects, respectively.

**Fig. 2:**
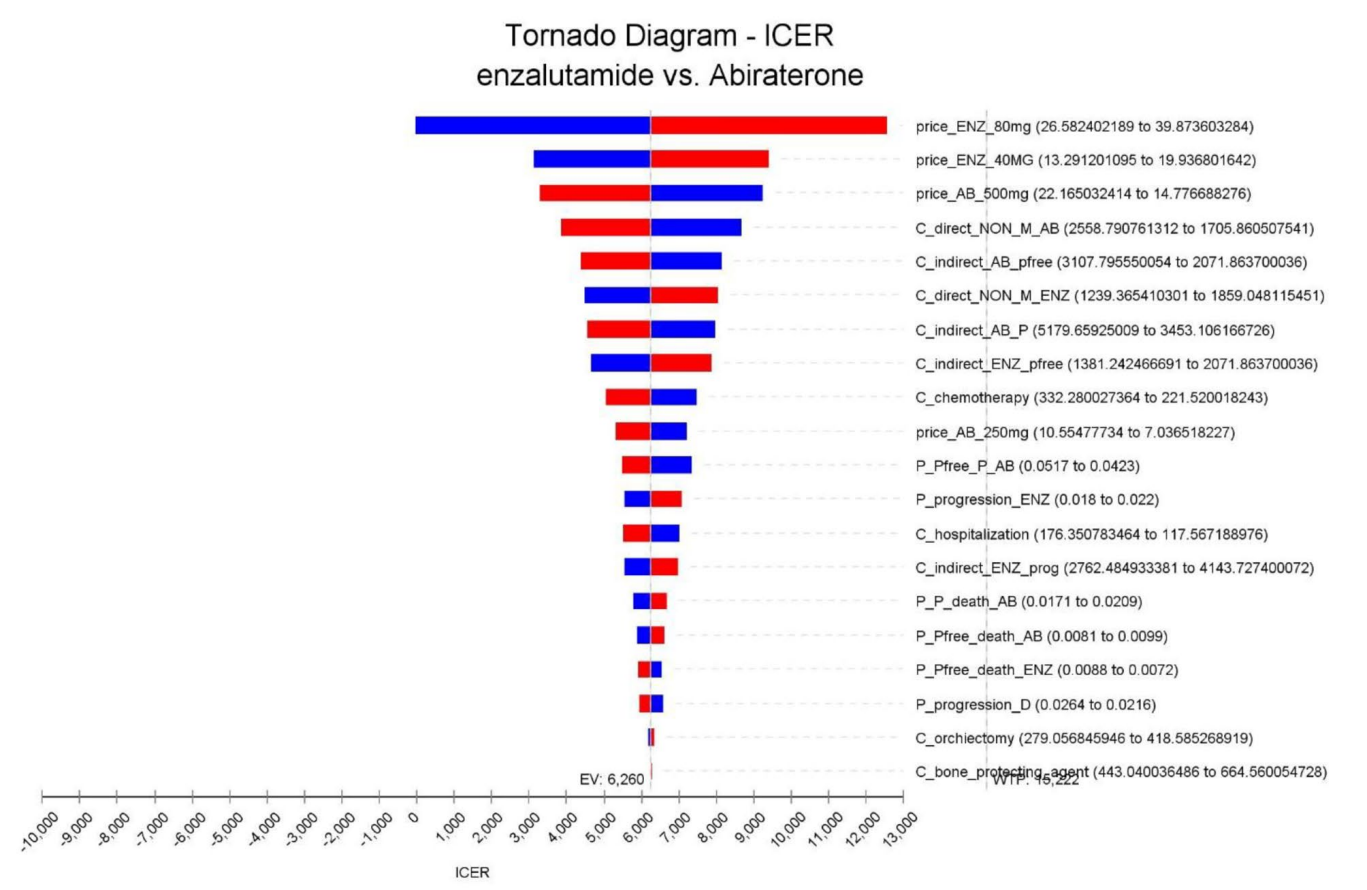
Tornado chart of the deterministic analyses

**Fig. 3:**
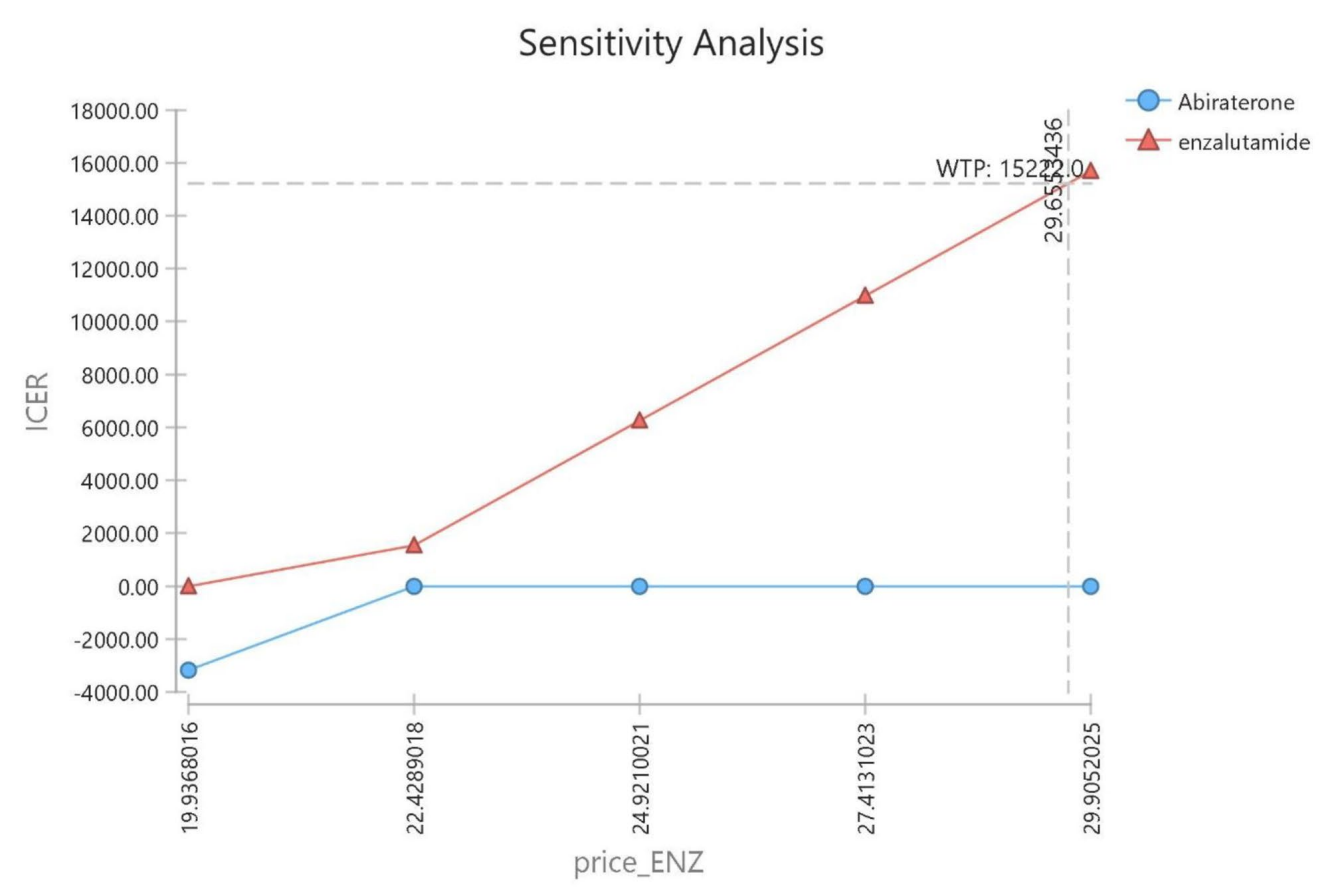
Chart of the one-way analyses

The ICER calculated by changing the determined ranges of the study parameters is shown as a tornado diagram in Fig. 2. On the other hand, as observed in Fig. 3, the one-way sensitivity analysis of the price of enzalutamide showed that the maximum range of cost-effectiveness of this drug would be up to $ 29.

**Fig. 4:**
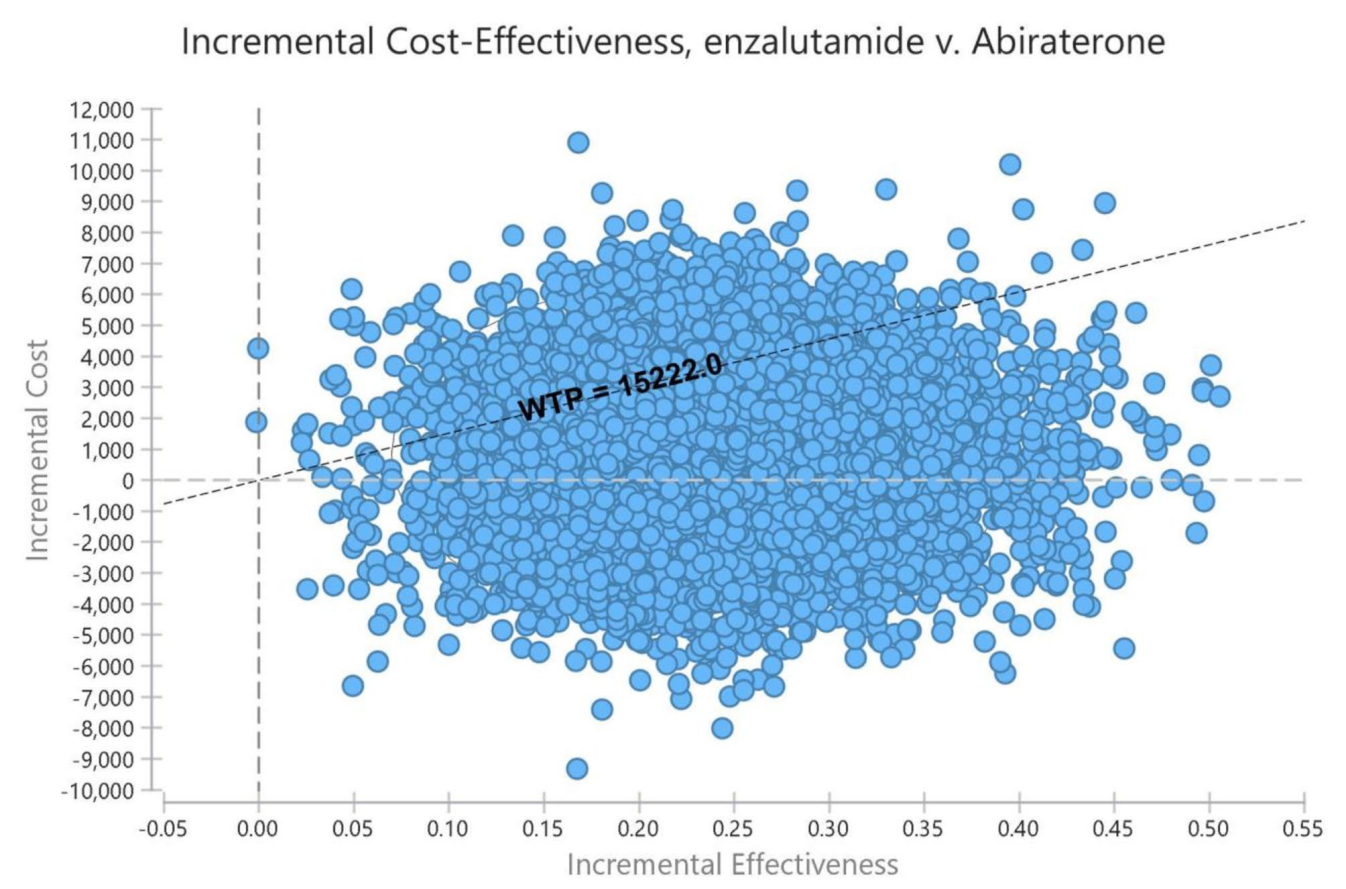
Scatter plot of PSA

The probabilistic sensitivity analysis based on the Monte Carlo simulation indicated that in 39% of the cases, ICER points were in the first quarter and below the threshold, implying higher cost and effectiveness of Enzalutamide, and in the second quarter in 50% of the cases, implying lower cost and greater effectiveness of enzalutamide compared to Abiraterone. (Fig. 4)

### Budget impact analysis

The results of the budget impact analysis are shown in Table 3. With an assumed market share of approximately 5% for enzalutamide in the first year, the budget impact would be $410,817in that year. With an enzalutamide share of 25% in the fifth year, the budget impact would be $2,154,470as well. The impact of the accumulated budget on the health system during 5 years was estimated at $6,362,127.

**Table 3:** The results of the budget impact analysis

Year	2024	2025	2026	2027	2028
Iran population	87,406,253	88,455,128	89,516,589	90,590,788	91,677,878
men population	43,703,126	44,227,564	44,758,295	45,295,394	45,838,939
mCRPC patients	3,846	3,892	3,939	3,986	4,034
enzalutamide market share	0.05	0.1	0.15	0.2	0.25
Scenario 1(without Enzalutamide)	39,055,041	39,523,702	39,997,986	40,477,962	40,963,697
Scenario 2(with Enzalutamide)	39,465,858	40,355,195	41,260,194	42,181,101	43,118,168
Financial impact	410,817	831,493	1,262,208	1,703,139	2,154,470

## Discussion

In this study, we evaluated the cost-effectiveness of enzalutamide and abiraterone in patients with castration-resistant metastatic prostate cancer. Based on our investigations, the cost-effectiveness of these two treatments in Iran had not been determined before.

According to the results of the analyses conducted in the present study, treatment with enzalutamide was more effective compared to abiraterone (1.02 vs. 0.84 QALY). However, the results of the previous studies on the effectiveness of enzalutamide and abiraterone regimens in patients with metastatic prostate cancer resistant to castration were different. For example, it had been reported in a scoping review that due to the different outcomes and toxicities of these two treatment regimens based on the specific factors of the patients, clinicians treated the patients in a personalized way. The study reported more neurological complications and fatigue in patients treated with Enzalutamide, and higher cardiac complications in those treated with Abiraterone [37].

However, the results of the present study were consistent with a systematic review of clinical trials that had examined overall survival (OS) indicators and radiographic progression-free survival (rPFS). The results indicated that both drugs significantly reduced mortality and radiographic progression-free survival compared to placeboes. Nevertheless, the results did not show a significant difference between the mortality rates. However, enzalutamide was introduced as the preferred treatment regimen regarding the radiographic progression-free survival [13].

The results of the sensitivity analysis in the present study confirmed the findings of the study by Schultz et al., who stated that although treatment with enzalutamide had higher complications, it was associated with fewer outpatient visits and hospitalizations compared to patients treated with Abiraterone. One of the possible reasons could be the frequent use of corticosteroids and subsequent infections in these patients compared to other treatment regimens [38].

Our analysis regarding the direct and indirect costs of the two treatment regimens indicated that both direct non-medical cost (1,549 vs. 2,132) and indirect medical cost were lower in the enzalutamide treatment regimen (5,179 vs. 6,906). However, direct medical costs were higher in the enzalutamide group than in the abiraterone (17,541 vs. 16,408). In recent years, with the introduction of new drug approaches for the treatment of MCRPC, the potential economic burden of treating this disease has subsequently increased. Schultz and Ramaswamy conducted studies on abiraterone and enzalutamide treatment costs; the former researcher indicated lower monthly costs for abiraterone than enzalutamide ($5,756 for abiraterone vs. $6,879) [38], but the latter stated that the monthly costs of enzalutamide were lower than those of abiraterone ($3,953 vs. $4,663; *p* = 0.0182).

Based on the results of Ramaswamy et al.‘s study, the cost of enzalutamide was $2,666 less than that of abiraterone in a one-year horizon [39]. Similar to the present study, other studies also reported higher costs of Enzalutamide. In their study, Salgado and Elias took into account the duration of treatment, the level of adherence, and the number of patients under treatment, and reported lower treatment costs regarding abiraterone acetate plus prednisone than enzalutamide in mCRPC patients previously treated with docetaxel [40, 41]. On the contrary, Schultz et al. took into account the overall consumption of health services by the patients and reported lower enzalutamide costs due to less use of health services (outpatient, inpatient, and emergency) [38, 39]. The important point is that most of these results were obtained by indirect comparisons of the mentioned drugs [42].

The present study showed that the average costs per QALY obtained in the enzalutamide and abiraterone treatment regimens were 17,197 and 19,533, respectively, indicating higher costs and QALYs in the former treatment regimen. On the other hand, according to the considered threshold of one time GDP in the present study ($18,261 per QALY), the ICER was 6,260, which was less than the threshold. Therefore, enzalutamide treatment regimen was more cost-effective and preferable compared to Abiraterone. This finding was in line with the economic analyses by Wilson et al [43] and Okumura et al [32]. According to the 5-year budget impact analysis, the economic burden of enzalutamide treatment regimen would be $6,362,127. The increase in treatment regimen costs in case of access to enzalutamide would be compensated to some extent by the reduction of post-advancement costs. Based on the results of the sensitivity analysis, the model was most sensitive to the parameters of enzalutamide price, abiraterone price, direct non-medical costs, and indirect costs. With a change in the parameters, the ICER remained in the lower range than the threshold. The PSA results suggested that the enzalutamide regimen was cost-effective and superior to abiraterone in 89% of simulations in the patients.

### Limitations

The present study had several limitations. First, clinical evidence was not obtained from the Iranian patients with mCRPC due to the lack of enzalutamide in Iran, and to date, no studies had been conducted on rPFS and OS for enzalutamide vs. Abiraterone. Therefore, valid studies were used to collect the efficacy and safety information. Second, given that clinical evidence on enzalutamide and abiraterone was not obtained from a direct comparison (as in head-to-head clinical studies), the heterogeneity of each study might have affected the results of the analyses. Third, due to the lack of evidence, the rate of treatment discontinuation related to side effects was not considered in our analysis.

## Conclusion

Based on the results of the present study, the enzalutamide treatment was more effective and costly compared to the abiraterone regimen, but compared to the threshold limit, this treatment was preferable for mCRPC patients. Considering the high costs of treating mCRPC patients, it is suggested that further studies be conducted on direct costs of the complications of these treatment regimens.

## Data Availability

The datasets used and/or analyzed during the current study are available from the corresponding author on reasonable request.
